# Chronic periodontitis and community-acquired pneumonia: a population-based cohort study

**DOI:** 10.1186/s12890-019-1017-1

**Published:** 2019-12-30

**Authors:** Seon-Jip Kim, Kyuwoong Kim, Seulggie Choi, Jooyoung Chang, Sung Min Kim, Sang Min Park, Hyun-Jae Cho

**Affiliations:** 1 Department of Preventive Dentistry & Public Oral Health, School of Dentistry and Dental Research Institute, Seoul National University, 101 Daehakro, Jongno-gu, Seoul, 03080 Republic of Korea; 2 Department of Biomedical Sciences, Seoul National University Graduate School, Seoul, 03080 Republic of Korea; 3 Department of Family Medicine, Seoul National University Hospital, Seoul, 03080 Republic of Korea

**Keywords:** Cohort studies, Epidemiology, Periodontal disease, Pneumonia

## Abstract

**Background:**

This study aimed to determine the association between chronic periodontitis (CP) and community-acquired pneumonia (CAP) according to CP severity in the Korean population based on the National Health Insurance Service database.

**Methods:**

Data from the National Health Insurance Service-National Health Screening Cohort (NHIS-HEALS), conducted from 2002 to 2013, were analyzed. A total of 363,541 participants were enrolled in this retrospective cohort study. Data on socio-demographic characteristics and CAP-related variables were collected. Participants were divided into 4 groups according to CP severity. Cox proportional hazards regression was performed after adjusting for sociodemographic and related covariates.

**Results:**

A total of 363,541 participants were included in the analysis. The number of CAP cases in the index period was 14,838 (4.1%). Among the 4 groups, the mean age was significantly higher in the severe CP group. The incidence rates of severe and non-severe CP were 5.68 and 4.99, respectively (per 10^3^ person-years). The hazard ratio for CAP was not significant in any of the models regardless of the presence or absence of CP. On stratification analysis by sex, smoking and Charlson comorbidity index, there were no significant differences between CAP and CP in any of the models.

**Conclusion:**

The results of this study show that CP may not be a potential risk factor for CAP.

## Background

Periodontal disease is a chronic inflammatory disease of the periodontal tissue that affects approximately 20 to 50% of the world’s population [1]. It is caused by colonization of periapical periodontal pathogens that cause destruction of the ligaments and alveolar bone supporting the teeth [2, 3]. Chronic periodontitis (CP) results in tooth loss due to an advanced inflammatory form of periodontal disease caused by microorganisms [4]. Research has suggested that periodontal disease is a risk factor for systemic disease, and studies have shown that periodontitis is associated with an overall increased risk of mortality [5, 6].

Pneumonia is one of the most common serious infections and causes significant morbidity and mortality in both healthy and vulnerable individuals [7]. Pneumonia is divided into two categories: hospital-acquired pneumonia (HAP) and community-acquired pneumonia (CAP). HAP refers to pneumonia that develops 48 h after admission or within 10 days after discharge from a hospital without incubation [8], and CAP refers to an acute infection of the lungs in individuals who have not been recently hospitalized and are not regularly exposed to the healthcare system [9]. According to data from the Korea National Statistical Office, pneumonia was the 10th leading cause of death in 2004, but ranked 4th after cancer, cardiovascular disease, and cerebrovascular disease in 2015 [10]. The main causes of mortality, which increased significantly from 10 years ago, were pneumonia (22.9 persons, 246.8%), heart disease (17.1 persons, 41.5%), and lung cancer (6.5 persons, 22.5%) [10]. Numerous researchers have shown an association between periodontitis and respiratory disease such as pneumonia [11–15].

Most previous studies on periodontal disease and pneumonia were case-control or cross-sectional studies and did not involve the general population, just elderly or vulnerable populations such as patients who have HAP or ventilator-associated pneumonia. Additionally, these studies were limited to HAP and not CAP. Moreover, the relationship between periodontal disease and CAP has yet to be established by longitudinal studies. This study aimed to analyze the relationship between CAP and CP using the Korean National Health Insurance Service-National Health Screening Cohort (NHIS-HEALS) data.

## Methods

### Study population

This was designed as a retrospective cohort study following participants from 2002 to 2013 and was conducted using the NHIS-HEALS data. The NHIS has provided compulsory social insurance for all Korean individuals since 1989, with an enrollment rate of nearly 98% [16]. The NHIS-HEALS cohort consisted of random samples representing approximately 500,000 individuals aged 40–79 years, which is equal to 10% of the total population between 2002 and 2003 [17]. The NHIS-HEALS includes eligibility and demographic information regarding health services and data on medical aid beneficiaries, medical bills, medical treatment, medical history, and prescriptions. The NHIS is responsible for paying premiums to medical institutions. The Institutional Review Board (IRB) of Seoul National University Hospital approved this study (IRB number, 1801–019-912), which is in compliance with the Declaration of Helsinki.

**Fig. 1:**
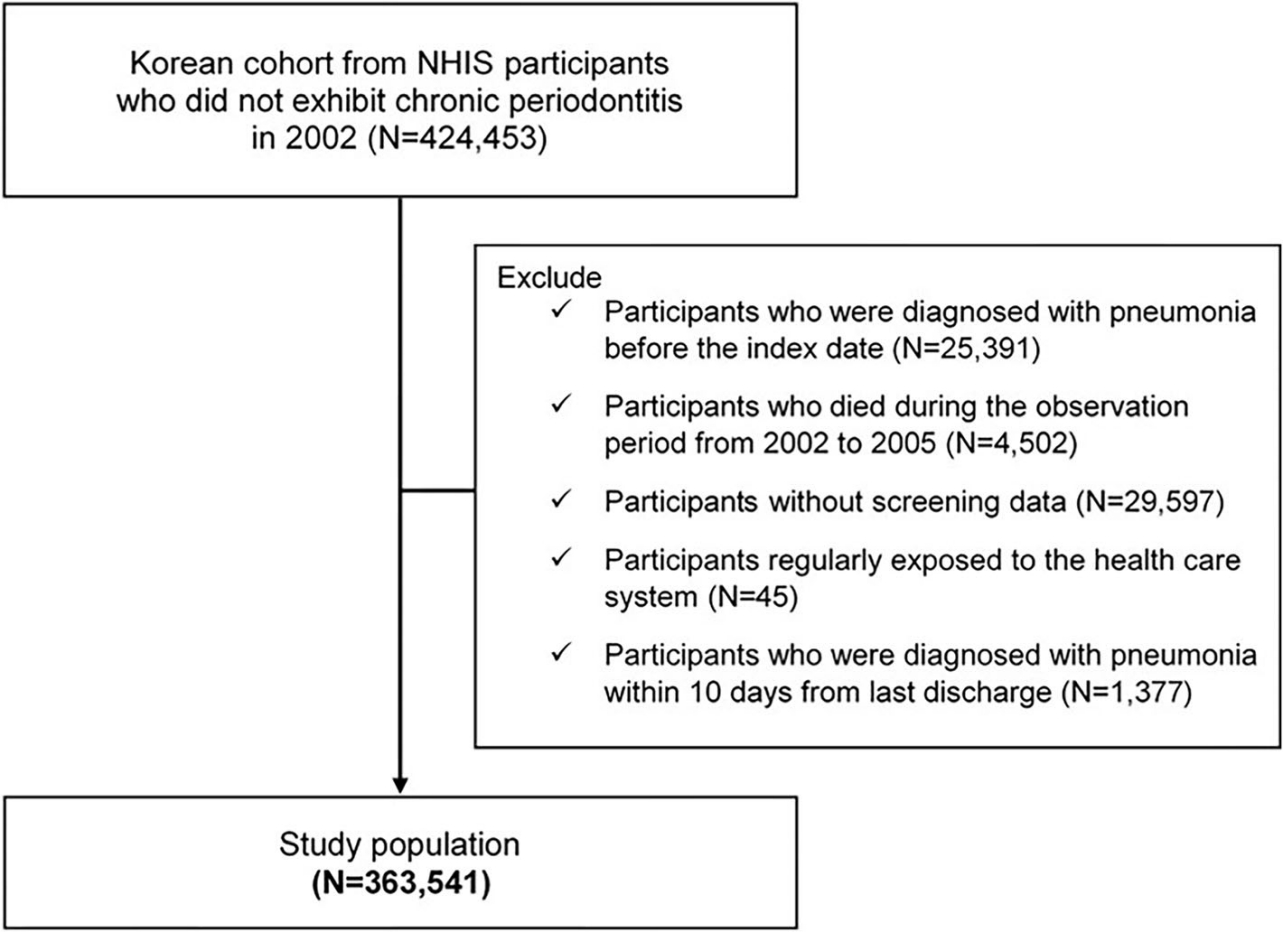
Flow chart of study population from the National Health Insurance Service-Health Screening Cohort database in the Republic of Korea

A total of 424,453 subjects were selected, excluding those who had CP in 2002. Among these individuals, 25,391 who were diagnosed with pneumonia before follow-up (index date, January 1, 2006) according to the International Classification of Diseases (ICD) codes (10th revision) by the World Health Organization for pneumonia were excluded from the study. A further 4628 participants who died and 29,597 participants with missing screening data were excluded. We also excluded those who had Problems related to care-provider dependency who were likely to be exposed to the healthcare system, such as those undergoing regular dialysis and those who living in nursing homes [18]. Finally, to exclude subjects who had HAP during the follow-up period, 1377 participants diagnosed with pneumonia within 10 days from last discharge were excluded (Fig. 1). The study population consisted of 363,541 participants (190,933 men and 172,608 women).

### Data collection

The study population was divided into 4 groups according to the severity of periodontitis (non-CP, mild CP, moderate CP, and severe CP). CP was defined by the ICD-10 codes (K05.30, K05.31, K05.32, K05.38, K05.39), and severity of periodontal disease was classified according to periodontal surgery performance. Patients with periodontitis who underwent scaling and root planing were classified into the mild CP group, while patients who only received subgingival curettage were classified into the moderate periodontitis group. Patients who underwent tooth extraction and severe dental treatment such as periodontal flap operation, bone graft for alveolar bone defects, and guided tissue regeneration, were assigned to the severe CP group (Table 1).

**Table 1:** Severity of chronic periodontitis and community-acquired pneumonia

Chronic periodontitis	ICD-10 (K05.30, K05.31, K05.32, K05.38, or K05.39)	Treatment code
Severity	Treatment	
Mild	Scaling	U2232, U2233
	Root planing	U2240
Moderate	Subgingival curettage	U1010
Severe	Tooth extraction	U4411, U4412
	Periodontal flap operation - simple	U1051, U1052
	Bone graft for alveolar bone defects	U1071, U1072
	Guided tissue regeneration	U1081, U1082, U1083
Pneumonia	ICD-10 (J12, J13, J14, J15, J16, J17, or J18)	Form of visit
Community-acquired pneumonia	Excluded Care involving dialysis (ICD-10, Z49) Dependence on renal dialysis (ICD-10, Z99.2) Problems related to care-provider dependency (ICD-10, Z74) Pneumonia within 10 days from the last discharge	Admission

The main outcome of this study was admission due to CAP from January 1, 2006 to December 31, 2013. Hospital admission due to pneumonia was defined as hospitalization for ≥1 day. From the index date, participants were followed until the date of admission for pneumonia or until the day when the main outcome did not occur and the patient survived or died on the way. Covariates were based on the data before the index year and comprised age, sex, household income (quartile), body mass index (BMI, kg/m2), smoking status (never, former, and current), alcohol consumption (< 3, and ≥ 3 times per week), physical activity (< 3, and ≥ 3 times per week), total cholesterol level (mg/dL), and Charlson comorbidity index (CCI, 0, 1–2, and ≥ 3). CCI is the most widely used tool for predicting the prognosis of comorbid disease [19].

### Statistical analysis

For each CP group, the association between CP and CAP was assessed with an adjusted Cox proportional hazards regression model. Among the covariates, age, BMI, total cholesterol level, and fasting serum glucose level were considered continuous variables. Participants who did not develop CP and CAP were included as the reference group. To identify potential subgroups with significant associations between CP and pneumonia, we performed stratification analysis that included sex, smoking status, and CCI. Four Cox proportional hazards regression models were hierarchically designed to analyze the influence of each group of covariates. All data collection and statistical analysis in this study were performed using SAS 9.4 (SAS Institute, Cary, NC, USA). Statistical significance in this study was defined as a *P*-value < 0.05.

## Results

A total of 363,541 participants were followed for an average of 7.6 years (standard deviation, 1.3), resulting in 2,782,453 person-years. Table 2 shows the baseline characteristics of all study participants according to the severity of periodontal disease. Among 363,541 participants, 30.1% (*n* = 109,322) had CP. The number of CAP cases during the follow-up period was 14,838 (4.1%). The mean age of the participants was 57.8 years, and the mean age of the severe CP group was significantly higher than that of the Non-CP group. Table 3 shows the multivariable association results from Cox proportional hazard regression analyses between CP and CAP after adjustment for age, sex, household income, smoking status, alcohol consumption, physical exercise, CCI, BMI, fasting serum glucose level, and total cholesterol level. In those with CP, the severe CP group had the highest number of cases (*n* = 2798), while the moderate group (*n* = 568). The hazard ratio (HR) for CAP was not significant in any of the models regardless of the presence or absence of CP. Table 4 shows the multivariable association results from Cox proportional hazard regression analyses between severe CP and CAP after adjustment for age, sex, household income, smoking status, alcohol consumption, physical exercise, CCI, BMI, fasting serum glucose level, and total cholesterol level. There were no significant associations in any model (Model 4 HR, 1.00; 95% confidence interval, 0.96–1.04). Table 5 shows subgroup analysis of the association between CP and the risk of pneumonia. The results in all subgroups stratified by sex and BMI were similar to the main results. There was also no significant difference in smoking status, which is commonly accepted as a strong risk factor for CAP.

**Table 2:** Characteristics of participants according to chronic periodontitis severity

	Non-CP	Chronic periodontitis			
		Mild CP	Moderate CP	Severe CP	*p* value
Number of subjects, n, (%)	254,219 (69.9)	31,104 (8.6)	16,303 (4.5)	61,915 (17.0)	
Number of CAP cases, n, (%)	10,349 (69.7)	1123 (7.5)	568 (3.8)	2798 (18.9)	
Age, years, mean (SD)	57.6 (8.8)	57.3 (8.2)	56.3 (7.6)	59.1 (8.7)	< 0.001
Sex, %					
Male	52.6	53.0	52.8	51.8	< 0.001
Female	47.4	47.0	47.2	48.2	
Household income, quartile, %					
1st (highest)	25.0	24.9	24.6	24.4	0.067
2nd	25.3	25.6	25.5	25.3	
3rd	21.7	21.8	22.2	22.3	
4th (lowest)	28.0	27.7	27.7	28.0	
Smoking status, %					
Never	70.7	70.6	70.1	71.6	< 0.001
Former	8.7	8.7	9.1	8.6	
Current	20.6	20.7	20.8	19.8	
Alcohol consumption, per week, %					
< 3 times	88.9	89.0	88.8	88.8	0.811
≥ 3 times	11.1	11.0	11.2	11.2	
Physical exercise, per week, %					
< 3 times	78.4	78.3	78.0	78.5	0.556
≥ 3 times	21.6	21.7	22.0	21.5	
CCI, %					
0	34.8	35.0	36.5	32.8	< 0.001
1–2	50.8	50.9	50.3	51.4	
≥ 3	14.4	14.1	13.2	15.8	
BMI, kg/m^2^, mean (SD)	24.0 (3.0)	24.1 (2.9)	24.1 (2.9)	24.0 (3.0)	0.022
Fasting serum glucose level, mg/dL, mean (SD)	99.4 (32.4)	99.5 (32.6)	99.1 (32.8)	99.9(32.9)	0.002
Total cholesterol level, mg/dL, mean (SD)	200.4 (37.9)	201.0 (37.9)	200.4 (37.7)	200.7 (38.3)	0.079

Continuous variables are expressed as mean (SD), and categorical variables as %

Analysis of variance for continuous variables and Chi-square test for categorical variables

*CAP* community-acquired pneumonia, *SD* standard deviation, *CCI* Charlson comorbidity index, *BMI* body mass index

**Table 3:** Hazard ratio for community-acquired pneumonia according to severity of chronic periodontitis

Community-acquired pneumonia	Non-CP	Severity of CP		
		Mild	Moderate	Severe
Events	10,349	1123	568	2798
Model 1				
HR (95% CI)	1.00 (reference)	0.95 (0.89–1.01)	1.03 (0.94–1.12)	1.00 (0.96–1.04)
Model 2				
HR (95% CI)	1.00 (reference)	0.95 (0.89–1.00)	1.03 (0.94–1.12)	1.00 (0.96–1.04)
Model 3				
HR (95% CI)	1.00 (reference)	0.95 (0.89–1.01)	1.03 (0.94–1.12)	1.00 (0.96–1.04)
Model 4				
HR (95% CI)	1.00 (reference)	0.95 (0.89–1.01)	1.03 (0.94–1.12)	1.00 (0.96–1.04)

Model 1 was adjusted for age and sex

Model 2 was adjusted for age, sex, and household income

Model 3 was adjusted for age, sex, household income, smoking status, alcohol consumption, and physical exercise. Model 4 was adjusted for age, sex, household income, smoking status, alcohol consumption, physical exercise, Charlson comorbidity index, body mass index, fasting serum glucose level, and total cholesterol level

*CP* chronic periodontitis, *HR* hazard ratio, *CI* confidence interval

**Table 4:** Hazard ratio for community-acquired pneumonia according to severe chronic periodontitis and non-severe chronic periodontitis

Community-acquired pneumonia	Presence of severe CP	
	Non-severe CP^a^	Severe CP
Events	12,040	2798
Incidence rate^b^ (95% CI)	4.99 (4.26–6.00)	5.68(4.86–6.83)
Model 1		
HR (95% CI)	1.00 (reference)	1.00 (0.96–1.05)
Model 2		
HR (95% CI)	1.00 (reference)	1.00 (0.96–1.04)
Model 3		
HR (95% CI)	1.00 (reference)	1.00 (0.96–1.04)
Model 4		
HR (95% CI)	1.00 (reference)	1.00 (0.96–1.04)

Model 1 was adjusted for age and sex. Model 2 was adjusted for age, sex, and household income. Model 3 was adjusted for age, sex, house income, smoking status, alcohol consumption, and physical exercise. Model 4 was adjusted for age, sex, household income, smoking status, alcohol consumption, physical exercise, Charlson comorbidity index, body mass index, fasting serum glucose level, and total cholesterol level

*CP* chronic periodontitis, *HR* hazard ratio, *CI* confidence interval

^a^Non-severe CP includes non-CP, mild CP, and moderate CP

^b^Per 10^3^ person-years

**Table 5:** Subgroup analysis of the association between chronic periodontitis and the risk of community-acquired pneumonia

	Non-severe CP^a^	Severe CP
Community-acquired pneumonia		
Men		
Number of cases	6872	1559
HR (95% CI)	1.00	0.98(0.92–1.04)
Women		
Number of cases	5168	1239
HR (95% CI)	1.00	1.03(0.97–1.10)
Smoker		
Number of cases	2908	667
HR (95% CI)	1.00	1.01(0.93–1.10)
Non-smoker		
Number of cases	8115	1896
HR (95% CI)	1.00	1.00(0.95–1.05)
CCI = 0		
Number of cases	2355	535
HR (95% CI)	1.00	1.01(0.92–1.11)
CCI = 1 or 2		
Number of cases	6417	1499
HR (95% CI)	1.00	1.00(0.96–1.04)
CCI ≥ 3		
Number of cases	3268	764
HR (95% CI)	1.00	0.97(0.91–1.02)

Hazard ratio analyzed by Cox proportional hazards regression analysis adjusted for age, sex, house income, physical activity, smoking status, alcohol consumption, body mass index, total cholesterol level, fasting serum glucose level, and Charlson comorbidity index

*CP* chronic periodontitis, *HR* hazard ratio, *CI* confidence interval, *CCI* Charlson comorbidity index

^a^Non-severe CP includes non-CP, mild CP, and moderate CP

## Discussion

As a prospective study on a large Korean adult population over a long follow-up period, this study has advantages. To the best of our knowledge, no cohort study has previously examined the association between admission due to pneumonia and CP.

CP was not found to significantly affect CAP, even after adjustment for important health characteristics. Our results were consistent with those of Brown’s study, which reported that CAP was not related to dental biofilms [7]. The main causative agents of CAP are *Streptococcus pneumoniae* and *Haemophilus influenzae*. CAP is also caused by the spread of viral bacterial pathogens such as *Mycoplasma pneumoniae*, *Chlamydia pneumoniae*, and *Legionella pneumophila*. *Porphyromonas gingivalis* and *Treponema denticola*, which are the bacteria involved in CP [20], and are not typically CAP-related bacteria.

Many studies have investigated periodontal disease as a risk factor for various types of pneumonia, including aspiration pneumonia, HAP, and ventilator-associated pneumonia [13, 21–26]. Aspiration of colonized pathogens has been proposed to be an important risk factor for pneumonia [11, 27–29]. de Melo Neto et al. demonstrated that moderate and severe CP were associated with CAP [30]; however, the study had a small sample size (140 patients) and was conducted for only 17 months. Moreover, the control group consisted of hospitalized patients and did not involve the general population. In contrast, this cohort study was the result of an 8-year follow-up of patients hospitalized with CAP according to the presence of CP, with the general population at baseline at relatively low risk of disease compared to the subjects of de Melo Neto’s study. This could explain the difference in our results and those of previous studies.

Several hypotheses have been posited to explain the likelihood of developing periodontal disease and several types of pneumonia. Periodontal disease with periodontal pockets promotes accumulation of dental plaque, which can promote the growth and reproduction of pathogenic bacteria. Various pathogenic bacteria have been found in patients with deep periodontal pockets [31, 32]. The association between periodontal disease and pneumonia may be due to colonization by pathogenic bacteria in the periodontal pocket, as inhalation of a pathogen is considered a risk factor for pneumonia [33, 34]. Oral pathogens can be aspirated into the lower airways, which results in favorable conditions for the development of pneumonia [35, 36]. Excessive production of inflammatory cytokines induced by the major pathogens of periodontal disease plays a significant role in pneumonia [37]. The enzymes in saliva on the surface of the oral mucosa of patients with periodontal disease may facilitate the adhesion of respiratory disease pathogens [38]. However, it is difficult to clarify the cause of CAP, unlike those in other types of pneumonia. Most studies that identified the causes of CAP were conducted at tertiary referral hospitals, which may not represent the general population. Despite considerable efforts, rarely can the cause of CAP be clearly determined, and more rigorous investigations are needed [9, 39–41].

Few studies have been conducted on oral health and pneumonia when distinguishing between CAP and aspiration pneumonia. CAP and aspiration pneumonia share common risk factors such as diabetes mellitus, malnutrition, alcohol consumption, smoking status, and aging; however, the major risk factors for CAP are chronic obstructive pulmonary disease, heart disease, chronic bronchitis, functional impairment, chronic renal failure, cancer, and human immunodeficiency virus [27, 28, 42]. The major risk factors for aspiration pneumonia are poor oral health, sputum suctioning, use of antipsychotic drugs, deterioration of swallowing function, dehydration, and dementia [43, 44]. Therefore, CAP and aspiration pneumonia involve different risk factors and underlying diseases. For this reason, elderly individuals are more susceptible to pneumonia, and the causes and prevention methods of aspiration pneumonia and CAP are different [45]. In the subgroup analysis (Table 5), important CAP risk factors, such as smoking status and CCI, were stratified. However, consistent with the main results of this study, no statistically significant relationships could be identified.

There were several limitations to this study, mostly stemming from the use of claims data based on ICD-10 and treatment codes. First, the definition of CP based on treatment codes could underestimate the actual number of CP cases, especially in the mild and moderate periodontal disease groups. Several people seldom visit dental clinics or hospital because of lack of awareness regarding oral health and economic reasons. However, codes associated with severe CP treatment such as tooth extraction and periodontal surgery can be reliably used as identifying variables because severe CP is typically more painful for patients than mild or moderate CP. Therefore, we dichotomized the 4 groups of CP in Table 3 into severe CP and non-severe CP, and the results were not altered (Table 4). A previous study in Taiwan also used ICD and treatment codes to classify CP severity [46]. Second, this study defined CP not by clinical attachment loss, but by treatment code. Clinical attachment loss is often used to diagnose periodontal disease, and this information was not available in the database of this study. However, periodontitis is diagnosed based on dental examinations, including periodontal examination, probing depth analysis, and radiographic checkups, which are covered by national insurance. Similarly, inadequate clinical information, such as chest radiographs, blood samples, and pulmonary gas exchange data, is a major limitation when defining CAP [47]. To evaluate the causality between CP and CAP, pneumonia diagnosis needs to be supported by specific pathogen information to assess the type and severity, so further studies involving specific laboratory data will be particularly informative on this topic. To overcome the limitations associated with these data, we attempted to avoid overestimation by setting strict definitions of the main exposure and outcome. Lastly, it is possible that the new operational definition of CP is inaccurate. As the severity of CP increases, it would be logical for the percentage of subjects to decrease, but the severe CP group had the largest number of subjects. However, in a recent study that classified the stages of periodontal disease, subjects who their had teeth removed were at high risk of periodontal disease [48]. Based on this study, subjects with extractions were also classified into the severe CP group in the present study. Although treatment procedure codes are not a formal basis for determining the severity of CP, we aimed to establish a new operational definition based on input from dental specialists.

## Conclusions

This study suggested that CP is not a potential risk factor for CAP. The etiology and causality between periodontal disease and CAP remain unclear, and little is known about the underlying mechanism. In future studies, collection of clinical and epidemiological evidence is needed using a prospective study design to better understand the relationship between oral health and CAP. This study can be a starting point for discussion of the relationship between CP and CAP.

## Data Availability

The data supporting the findings of this study are available from National Health Insurance Sharing Service (https://nhiss.nhis.or.kr/bd/ab/bdaba000eng.do) in Korea, but are not publicly available due to restrictions on the availability of the materials used under the permission of this study.

## Abbreviations

BMI: Body mass index
CAP: Community-acquired pneumonia
CCI: Charlson comorbidity index
CI: Confidence interval
CP: Chronic periodontitis
HAP: Hospital-acquired pneumonia
HR: Hazard ratio
ICD: International Classification of Diseases
IRB: Institutional review board
NHIS-HEALS: National Health Insurance Service-National Health Screening Cohort
SD: Standard deviation
